# Changes in the gut microbiota of cloned and non-cloned control pigs during development of obesity: gut microbiota during development of obesity in cloned pigs

**DOI:** 10.1186/1471-2180-13-30

**Published:** 2013-02-07

**Authors:** Rebecca Pedersen, Anders Daniel Andersen, Lars Mølbak, Jan Stagsted, Mette Boye

**Affiliations:** 1 National Veterinary Institute, Technical University of Denmark, Bülowsvej 27, Frederiksberg C, 1870, Denmark; 2 Department of Food Science, University of Aarhus, Blichers Alle 20, Tjele, 8830, Denmark; 3 Present address: Chr. Hansen A/S, Bøge Alle 10-12, Hørsholm, DK-2970, Denmark

**Keywords:** Gut microbiota, Cloned pigs, Diet-induced obesity, Bacterial diversity, *Bacteroidetes*, *Firmicutes*

## Abstract

**Background:**

Obesity induced by a high-caloric diet has previously been associated with changes in the gut microbiota in mice and in humans. In this study, pigs were cloned to minimize genetic and biological variation among the animals with the aim of developing a controlled metabolomic model suitable for a diet-intervention study. Cloning of pigs may be an attractive way to reduce genetic influences when investigating the effect of diet and obesity on different physiological sites. The aim of this study was to assess and compare the changes in the composition of the gut microbiota of cloned vs. non-cloned pigs during development of obesity by a high-fat/high-caloric diet. Furthermore, we investigated the association between diet-induced obesity and the relative abundance of the phyla *Firmicutes* and *Bacteroidetes* in the fecal-microbiota. The fecal microbiota from obese cloned (n = 5) and non-cloned control pigs (n= 6) was investigated biweekly over a period of 136 days, by terminal restriction fragment length polymorphism (T-RFLP) and quantitative real time PCR (qPCR).

**Results:**

A positive correlation was observed between body-weight at endpoint and percent body-fat in cloned (r=0.9, *P*<0.0001) and in non-cloned control pigs (r=0.9, *P*<0.0001). Shannon Weaver and principal component analysis (PCA) of the terminal restriction fragments (T-RFs) revealed no differences in the bacterial composition or variability of the fecal microbiota between the cloned pigs or between cloned and non-cloned control pigs. Body-weight correlated positively with the relative abundance of *Firmicutes* in both cloned (r=0.37; *P*<0.02) and non cloned-control pigs (r=0.45; *P*<0.006), and negatively with the abundance of *Bacteroidetes* in cloned pigs (r=−0.33, *P*<0.04), but not in the non-cloned control pigs.

**Conclusion:**

The cloned pigs did not have reduced inter-individual variation as compared to non-cloned pigs in regard to their gut microbiota in neither the obese nor the lean state. Diet-induced obesity was associated with an increase in the relative abundance of *Firmicutes* over time. Our results suggest that cloned pigs are not a more suitable animal model for gut microbiota-obesity related studies than non-cloned pigs. This study is the first to evaluate if cloned pigs provide a better animal model than conventional pigs in diet-intervention, obesity and gut microbiota research.

## Background

Obesity and its associated morbidities have become an increasing problem in many countries around the world. While traditionally regarded as primarily a question of a sedentary lifestyle in which energy intake exceeds energy expenditure, new studies also point to the composition of the intestinal microbiota as a potentially contributing factor. In studies of diet-induced obesity and its association with the gut microbiota, it may be preferable to eliminate the influence of host genotype on the composition of the gut microbiota by choosing genetically identical animals. Some early investigations comparing the composition of the microbiota in human mono-zygotic twins (MZ) with di-zygotic twins (DZ) reported that the host genome was influencing the microbial composition in the gut [1,2]. A similar study based on 16S rRNA gene analysis indicated that bacterial community in human MZ twins was slightly more similar than in unrelated individuals [3] suggesting that genetically identical individuals harbor a similar gut microbiota. In a more recent study on the relationship between gut microbiota, diet and genetic influences in mice, the authors stated that the changes in gut microbiota were unrelated to genetically induced obesity and were merely due to high-fat (HF) diet [4]. Therefore, the influence of the host genome on the gut microbiota currently remains controversial.

When choosing an animal model for studying human diseases, it is important to choose animals that physiologically resemble humans. Pigs are good models for humans, primarily due to close resemblance of their anatomy and physiology of the digestive system and because pigs are omnivorous like humans [5,6]. Consequently, pigs are widely used in studies of human lifestyle-related diseases such as diabetes, cardiovascular disease and metabolic syndrome [7,8]. Using cloned pigs in obesity-related studies could provide a more homogenous experimental model, hence the cloning in this study was performed to minimize genetic influences and thereby reduce inter-individual variation [9].

One of the main focuses of obesity-related gut microbial studies have been to identify groups of bacteria that are correlated with the obese state, and initially the relative abundance of *Bacteroidetes* and *Firmicutes* in the gut microbiota was linked to obesity. In pigs, as in humans [10] and other mammals [11], the two main phyla of bacteria in the gut microbiota are *Bacteroidetes* and *Firmicutes*[12,13]. Previous studies have reported a greater proportion of *Firmicutes* in obese mice [14] when compared with their leaner counterparts and a reduced ratio of *Firmicutes* to *Bacteroidetes* in a small group of obese humans on a weight loss regimen [15]. A similar result in a study of lean and obese pigs revealed a negative correlation between percentage of *Bacteroidetes* and body-weight [16]. Furthermore, a fluorescence in situ hybridization (FISH)-based study on obese adolescents during weight loss regimens showed a decrease in the phylum *Firmicutes*[17]. However several studies suggest a decrease in ratio of *Firmicutes* to *Bacteroidetes* in obese and overweight subjects [18] and suggest diet to be a contributing factor in shaping the gut microbial community and not the bacterial proportions [19,20]. Other observations in humans, suggest obesity to be associated with a lower bacterial diversity [3], while other studies showed no difference in the abundance of bacteria in the gut microbiota between lean and obese individuals that were on weight maintaining diet [21]. Hence this putative relationship between obesity, diet and specific phyla of bacteria in the gut microbiota is still controversial and there are few studies on the association between the gut microbiota and obesity during the development of obesity. Therefore, the focus of this paper was to investigate the gut microbiota in cloned pigs compared with non-cloned control pigs and to further elucidate if diet-induced obesity over time is associated with changes in the gut microbiota. We hypothesized that the composition of the gut microbiota would be more similar among the cloned pigs compared to non-cloned controls. The second hypothesis was that weight-gain would be related to an increase in the ratio of *Firmicutes* to *Bacteroidetes* as well as a decrease in the diversity of the gut microbiota. We therefore investigated the changes in the gut microbiota of cloned and control pigs beginning with lean pigs during a period of 136 days on a high-fat/high-caloric (HF/high-caloric) diet.

## Methods

### Animals

The animals for this experiment were pigs of similar genotype of Danish Landrace and Yorkshire. Six female siblings from a normal litter (the control group) (75% Landrace x 25% Yorkshire) were obtained after standard artificial insemination followed by caesarian section. The cloning experiments were performed using donor cells obtained from a 65% Landrace x 35% Yorkshire sow as described previously [9]. The cloned embryos were then transferred surgically to surrogate sows (recipients) five to six days after cloning [9]. Two surrogate sows gave birth to five live female clones by caesarean section. Pigs were reared in the experimental stables at University of Aarhus (Tjele, Denmark). All the experimental animal studies were approved by the Danish Animal Experimental Committee.

### Experimental set up and sample collection

The pigs in the experiment were weaned at 28 days of age and subsequently fed a standard pig-diet with an energy distribution of 18.5% protein, 7.9% fat, 72.4% carbohydrate and 1.2% fiber, for approximately 61 days. During this post weaning period animals from the same litter were housed together in the same stable. At 96 days (cloned pigs) and 89 days (non-cloned controls) of age (baseline), the pigs were transferred to facilities for individual housing and fed a wheat-based HF/high-caloric diet consisting of 19.5% protein, 27% fat, 53% carbohydrates and 0.5% fiber [22] with *ad libitum* access to the feed in order to induce obesity. The feed was weighed before and after feeding and the pigs were maintained on this diet for a period of 136 days until they were euthanized. The cloned and non-cloned control pigs were weighed biweekly starting a day prior to switch to HF/high-caloric feed and the body-fat composition of the animals was measured by computed tomography (CT) scan at the end of the experiment. During this period, fresh feces collected biweekly were snap-frozen in liquid nitrogen and stored at −20°C until later analyses.

### Terminal restriction fragment length polymorphism (T-RFLP)

The fecal microbiota from all the pigs were analyzed by terminal restriction fragment length polymorphism (T-RFLP) fingerprint profiles as described previously [23]. In brief, DNA was extracted from 200 mg feces by using the QIAamp DNA Stool Mini Kit (Qiagen, Hilden, Germany) according to manufacturer’s instructions, with an additional step of bead beating in order to disrupt the cell wall of Gram-positive bacteria. The concentrations of DNA were measured in each sample by a spectrophotometer and adjusted to 5 ng μl^-1^ (NanoDrop Technologies,Wilmington, DE, USA). Amplification of 16S rRNA gene DNA were performed in duplicates by using 16S rRNA gene DNA bacterial specific primers, Eub-8fm (5’- AGAGTTTGATCMTGGCTCAG- 3’) labeled with 5´ FAM and Eub-926r (5-’CCGTCAATTCCTTTRAGTTT- 3’) (DNA Technology, Aarhus, Denmark) [23]. Each PCR mix contained 5 μl of 10x Fermentas Taq-buffer, 4 μl MgCl_2_, 2.0 μl deoxyribonucleotide triphosphate (dNTP), 0.5 μl Fermentas Taq-polymerase, 0.5 μl of each primer and 35.5 μl nuclease-free water and 5 ng μl^-1^ DNA (final concentration of 0.2 ng). The cycling conditions were: initial denaturation at 94°C for 6 minutes (min) followed by 32 cycles of denaturing at 94°C for 45 seconds (s), annealing at 56°C for 45 s, an extension step at 72°C for 2 min, and a final extension at 72°C for 10 min. The PCR products were subsequently verified by gel electrophoresis and purified by High Pure PCR Purification Kit (Roche Applied Sciences, Mannheim, Germany). The purified PCR product (200 ng) was digested with 2.0 μl of the restriction enzyme *HhaI* (Promega Corporation, Madison, USA) at 37°C for 3 h. Two μl of the digested PCR products, 10 μl formamide and 0.50 μl Megabase ET900-R Size Standard (GE Health Care, Buckinghamshire, UK) were mixed and run in duplicates on a capillary electrophoresis genetic analyzer (Genetic Analyzer 3130/3130xl, Applied Biosystems, Carlsberg, CA). The terminal restriction fragments (T-RFs), representing bacterial fragments in base pair (bp), were obtained and the analysis of T-RF profiles and alignment of T-RFs against an internal standard was performed using the BioNumerics software version 4.5 (Applied Maths, Kortrijk, Belgium).

T-RF fragments (range of 60–800 bp) with a difference less than two base pairs were considered identical. Only bands present in both duplicates were accepted as bacterial fragments from which the duplicate with the best intensity was chosen for microbial profiling. The obtained intensities of all T-RFs were imported into Microsoft Excel, and all intensities below 50 were removed. In each sample, the relative intensity of any given T-RF was calculated by dividing the intensity of the T-RF with the total intensity of all T-RFs in the sample. The most predominant T-RFs with a mean relative intensity above one percent were selected for all further analyses and procedures (except calculation of the diversity and similarity) and their identity was predicted *in silico*, performed in the MiCA on-line software [24] and Ribosomal Database Project Classifier (322.864 Good Quality, >1200) [25].

### T-RFLP statistical analysis

All T-RFs between 60 and 800 bp were imported into the statistical software programs Stata 11.0 (StataCorp, College Station, TX), Unscrambler version 9.8 (CAMO, Oslo, Norway) and Microsoft Excel sheets were used for further analyses. Principal component analysis (PCA) was used to explore group differences in the overall microbial communities both for comparisons between cloned pigs and non-cloned controls at the different sampling points and to investigate if samples from pigs with the largest weight-gain during the study period clustered together, irrespective of their genetic background. The latter was also investigated by relating the whole microbial community to the weight-gain at the different sampling points, involving all predominant T-RFs simultaneously in the models. For this purpose partial least square regression (PLS-R) was used, which is a supervised model, meaning in this case that the variation in the weight (gain) data is used to actively decompose the variation in the bacterial data. In both analyses, the T-RFs were standardized (centered and 1/SD) prior to the modeling phase to ensure that all of them would equally influence the models, and possible outliers were inspected visually and with *Hotelling T*^*2*^.

The diversity index was calculated as described previously [26]. In brief, the Shannon-Weaver index of diversity (*H*’) based on all of the initial T-RFs was used to determine the diversity of the bacterial fragments. Group comparisons of the diversity index in cloned versus non-cloned controls were calculated at each of the sampling points. As the Shannon-Weaver index was not normally distributed, Mann Whitney *U* test and Spearman correlation were applied. The *H*’ values are represented in figures as mean and error bars representing standard deviations (SD). Dice similarity between groups based on all the T-RFs were calculated in BioNumerics (Applied Maths, Kortrijk, Belgium) and the results are presented as mean values. T-RFs in the figures are presented as mean and standard error of the mean (SEM). A significant difference was considered when P-value was less than 0.05 (*P<0.05*).

### Fecal samples and bacterial strains for qPCR

The extracted DNA from the fecal samples used for the T-RFLP analyses were also analyzed by qPCR, but only samples taken monthly were chosen for qPCR analysis. However additional sampling points two weeks before the endpoint samples were also analyzed by qPCR. Three bacterial strains (*Clostridium perfringens* (NCTC 8449), *Odoribacter splanchnicus* (isolate DJF_B089) and *Escherichia coli* (ATCC 25922), representing the *Firmicutes* and *Bacteroidetes* phyla and general bacteria, respectively, and six randomly chosen extracted DNA samples (divided equally into clones and controls) were used to optimize the PCR conditions.

### qPCR primers and conditions

The 16S rRNA gene DNA primers for *Bacteroidetes* and *Firmicutes* used in this study were designed by Baccetti De Gregoris *et al.*[27] and conditions were optimized for the thermocycler used (Rotor-Gene Q Real Time PCR cycler (Qiagene)). The universal primer used in this study had an amplicon length of 147 bp (S-D-Bact-0907-a-S-20 5’-AAACTCAAAGGAATTGACGG-3’; S-D-Bact-1054-a-A-20 5-’ ACGAGCTGACGACAGCCATG-3’) [12]. The specific primer sets for *Bacteroidetes* (798cfbF 5’ CRAACAGGATTAGATACCCT’3 and cfb967R 5’ GGTAAGGTTCCTCGCGTAT ‘3) and *Firmicutes* (928F-Firm 5’ TGAAACTYAAAGGAATTGACG ‘3; 1040firmR, 5’ ACCATGCACCACCTGTC ‘3) had an amplicon length of 240 bp and 200 bp, respectively [27]. All qPCR reactions contained 12.5 μl of SYBR® Green JumpStart™ Taq ReadyMix™ without MgCl_2_ (Sigma-Aldrich, Copenhagen, Denmark), 0.3 μmol l^-1^ of each primer and 5 μl of template DNA adjusted to 5 ng μl^-1^. MgCl_2_ optimization was performed and a final concentration of 2.5 mM MgCl_2_ was chosen. The annealing temperature was optimized by using 16S rRNA gene DNA extracted from fecal samples and DNA extracted from different bacteria. Subsequently, all the primers and other PCR conditions were verified by conventional PCR and gel electrophoresis. A non template control (NTC) was included in each run. qPCR was performed with an initial denaturing step of 10 min at 95°C, 95°C for 30 s, 35 cycles of 56°C for 20 s and an elongation step of 72°C for 20 s. A melting curve analysis was performed after each run to detect any primer-dimers in each sample. The threshold cycle (*C*_*T*_) and calculated concentrations (copies μl^-1^) were determined automatically by the Rotor Gene software (Rotor-Gene Q 2.0.2 (Qiagene)).

### Analysis of data from qPCR

qPCR was performed to quantify relative abundance of the phyla *Bacteroidetes* and *Firmicutes,* respectively, present in each sample. The measured bacterial copy numbers of the 16S rRNA gene from bacteria belonging to the phylum *Bacteroidetes* and the phylum *Firmicutes* were calculated against 16S rRNA genes obtained from all bacteria and the relative abundance of the two phyla in each sample was subsequently calculated and statistically evaluated by Mann Whitney *U* test. Further correlation analyses were performed using Spearman correlation coefficient and *P* <0.05 was considered statistically significant. A standard curve was constructed for specific and universal primer sets and assays using tenfold serial dilutions of the extracted DNA from *C*. *perfringens*, *O. splanchnicus* and *E. coli* all DNA samples in the range 2.5 x10^2^ ng μL^-1^ to 2.5x10^-6^ ng μL^-1^. Furthermore, serial dilutions corresponding to the previously described dilutions of genomic DNA from two random samples were used to construct standard curves to further verify if PCR inhibitors were present in extracted DNA from fecal samples.

## Results

### Weight of the animals

**Figure 1:**
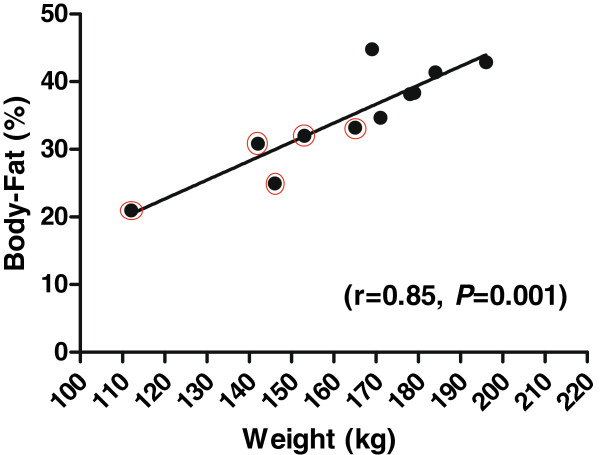
**Correlation between percent body-fat and body-weight (kg).** The correlation between percent body-fat and body-weight (kg) in all the pigs were calculated by Spearman correlation (r=0.85, *P=*0.0001). The red circles indicate the cloned pigs and the non-cloned pigs are indicated by plain black dots.

At baseline, just before the animals were transferred to the *ad libitum* high-fat (HF)/high-caloric diet, the cloned (96 days old) and non-cloned control (89 days old) pigs weighed 38 ± 4.1 kg (Mean ± SEM) and 37.9 ± 2.3 kg, respectively. Daily weight-gain in cloned pigs (n=5) was 0.78 ± 0.04 kg and in control pigs (n=6) 1.05 ± 0.03 kg, corresponding to a lower daily feed intake by cloned pigs than the controls. The clones weighed 143.6 ± 8.8 kg at the time they were euthanized (end point), compared to control pigs, which weighed significantly more (179.5 ± 4.0 kg) at the end of the study (difference of 35.9 kg, *P=*0.004). CT scanning of body fat showed that obese non-cloned control pigs had a higher average percentage of body-fat (41.1±1.3%) than obese cloned pigs (28.4 ± 2.3%, *P*=0.004). There was a positive correlation between body-fat percentage and body weight at the end of the diet-intervention study in non-cloned control pigs as well as in cloned pigs (r=0.85, *P*=0.0001) (Figure 1).

### The compositional diversity of the gut microbiota

**Figure 2:**
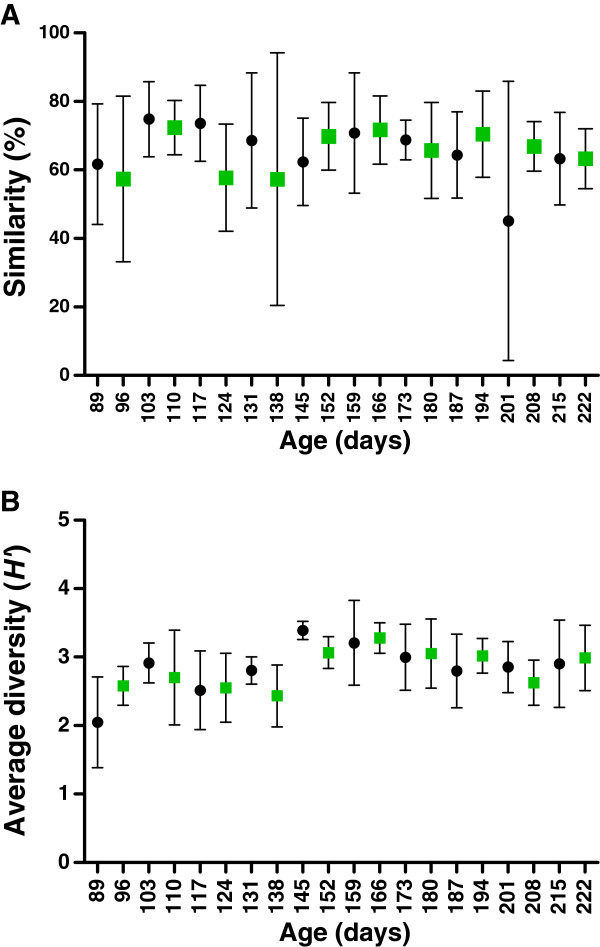
**Similarity (A) and diversity (B) of gut microbiota.** The similarity and diversity was calculated based on T-RFs (bp) at different age interval in non-cloned control pigs (● ) and cloned pigs (green square) by Dice similarity index and Shannon-Weaver index. Results are presented in mean and the error bars represent standard deviations (SD).

The PCA analysis of the overall composition of the gut microbiota in all animals did not reveal separate clustering of the T-RF profiles between the cloned pigs and the non-cloned controls. To test if the gut microbiota between cloned pigs was more similar than between non-cloned control pigs, a dice similarity score was calculated showing that the microbiota in cloned pigs was neither more uniform within the group nor more diverse compared to non-cloned control pigs (Figure 2A). Furthermore, there was no difference in Shannon-Weaver index between cloned and non-cloned control pigs at the start of diet-intervention (baseline), with Shannon-Weaver index (*H’*), *H’*=2.6 (2.3-2.8) and *H’*=1.7 (1.5-2.8), respectively. Within the control group, a slight increase (*P*=0.01) in the diversity of the gut microbiota was observed from baseline to end of diet-intervention (end point) (*H’*=3, 2.3-3.4), while no difference was observed in the cloned pig group (*H’*=3.3, 2.3-3.4) (Figure 2B). Furthermore, there was no correlation between diversity of microbial community as found by Shannon-Weaver index and weight-gain (Figure 2B).

**Figure 3:**
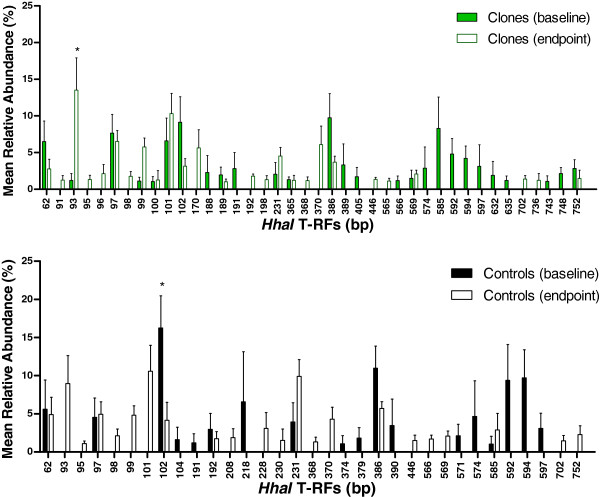
**The abundance of bacteria at baseline and endpoint.** Mean relative abundance of the most predominant T-RFs (>1%, bp) in the fecal samples of cloned pigs at baseline (green square) and endpoint (□ ) and in non-cloned control pigs at baseline (■ ) and endpoint (□ ). The error bars represent standard error of the mean (SEM).

The bacterial load (including all initial T-RFs between 60 and 800 bp) in the fecal microbiota of cloned pigs and non-cloned control pigs was similar throughout the intervention period, both at baseline and at endpoint (*P=*0.08 and *P=*0.3, respectively). In general, the T-RF profiles were similar in the cloned pigs and non-cloned pigs (Figure 3A and B). Both cloned pigs and non-cloned control pigs had 11 T-RFs with a relative abundance larger than one-percent in common at baseline and 17 T-RFs at endpoint (Figure 3A and B). There were several differences in T-RFs between the cloned pigs and non-cloned control pigs, however these were not significant (*P=*0.08).

In the non-cloned control group, one individual T-RF with a length of 102 bp was found higher at baseline compared to endpoint (*P=*0.04) (Figure 3B) and within the cloned pig group one T-RF (93 bp) was higher at endpoint than at baseline (*P=*0.01) (Figure 3A). At baseline in the non-cloned control group, the relative abundance of T-RF 93 bp was less than one percent and a significant increase in T-RF 93 bp from baseline to endpoint (*P=*0.005) was observed. The *in silico* analysis of the obtained T-RFs indicated that the T-RF 93 bp and T-RF 102 bp may be bacterial fragment belonging to the phylum *Bacteroidetes* (See Additional file 1).

At baseline a total of 47 T-RFs were present in the cloned pigs fecal microbiota while at endpoint there were 85 T-RFs present, indicating a more rich community at endpoint. At baseline 27 T-RFs with intensities of more than 1% are represented in Figure 3A. Together these 27 T-RFs represent 92% of the all the T-RFs present at baseline.

In non-cloned control pigs, a total of 42 T-RFs were present at baseline and 85 T-RFs were present at endpoint, again indicating an increase in T-RFs from baseline to endpoint. At baseline, only 18 T-RFs had intensities larger than 1%. These 18 T-RFs however, constituted 96% of all the T-RFs at baseline. At endpoint, there were 82 T-RFs present in fecal microbiota of non-cloned pigs of which only 22 T-RFs had intensities of more than 1% (Figure 3B). The possible identification of these T-RFs as found by *in silico* analysis, can be found in the supplementary material (See Additional file 1).

### Relative abundance of *Firmicutes* and *Bacteroidetes* in the gut microbiota by qPCR

There was no difference in the relative abundance of *Bacteroidetes* between cloned and non-cloned control pig at baseline (*P=*0.1) or at endpoint (*P=*0.9) and the same was observed for *Firmicutes* (baseline, *P=*0.8; endpoint, *P=*0.7).

**Figure 4:**
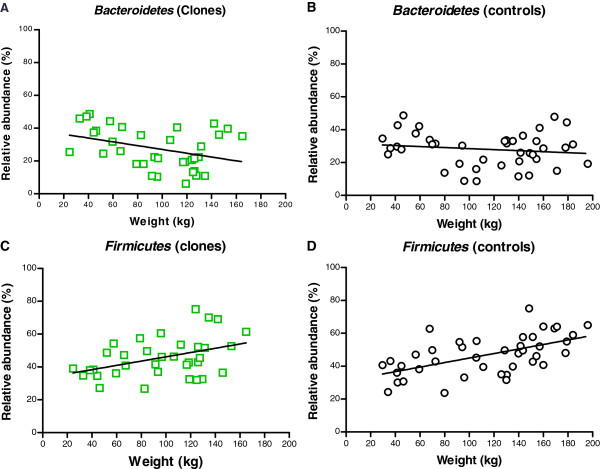
**Correlation between weight gain and relative abundance of *Bacteroidetes *and *Firmicutes*.** Correlation between weight gain and relative abundance of *Bacteroidetes* as calculated by Spearman correlation in cloned pigs (open green squares) (**A**) (r= −0.33, *P*<0.04) and non-cloned control pigs (○) (**B**) and correlation between weight-gain and relative abundance of *Firmicutes* in cloned pigs (open green squares) (**C**) (r= 0.37, *P*<0.02) and non-cloned control pigs (○) (**D**) (r=0.45, *P*<0.006).

In cloned pigs, a negative correlation was observed between weight-gain and relative abundance of *Bacteroidetes* (r= −0.33, *P*<0.04) (Figure 4A). A continuous and significant decrease (*P*<0.008) was observed in phylum *Bacteroidetes* from baseline and throughout the weight-gain period (Figure 4A) which then began to rise again by the time the pigs had an average weight of 118.9 ±3.2 kg until the animals were euthanized at endpoint.

In the non-cloned control pigs, there was a decrease in the relative abundance of *Bacteroidetes* from baseline (weight: 37.9 ± 2.3 kg) until the pigs weighed 95.5 ±3.9 kg, from which point the relative abundance of *Bacteroidetes* began to increase again until endpoint (Figure 4B). Subsequently, there was no significant difference in the relative abundance of *Bacteroidetes* at baseline and endpoint in the non-cloned pigs (Figure 4B).

In cloned pigs, an increase in relative abundance of *Firmicutes* was observed from baseline to endpoint (*P*<0.009) (Figure 4C) and the same was observed in non-cloned control pigs from baseline to endpoint (*P*<0.0001) (Figure 4D). This positive correlation between the weight-gain and the relative abundance of *Firmicutes* during the study period was observed both in cloned pigs (r= 0.37, *P*<0.02) and non-cloned control pigs (r=0.45, *P*<0.006) (Figure 4C and D, respectively). Additional figure shows the changes in the relative abundance of *Firmicutes* and *Bacteroidetes* during weight-gain (See Additional file 2).

## Discussion

In order to establish a better understanding of the underlying causes of obesity and the effect of obesity on different body sites, the cloned pigs and non-cloned control pigs employed for our study were also investigated in regard to their immunological [28], metabolomics [22] and phenotypic characters [9]. In this study, we investigated the gut microbiota of both cloned and non-cloned control pigs by T-RFLP and found that the gut microbiota within a group of five obese clones was neither more similar nor more diverse than the microbiota within a group of six obese non-cloned control pigs of the same sex and genetic background. The metabolomic phenotyping [9] of these obese cloned and non-cloned control pigs showed that the phenotype of the cloned pigs was different from the phenotype of non-cloned control pigs [9] and that the inter-individual variation amongst these cloned pigs was not less than the inter-individual variation of the non-cloned control pigs that were siblings [22]. Hence, based on these and the findings presented in the current paper it would appear that the cloned pigs do not have identical phenotypes or less inter-individual variation than conventional non-cloned pigs. One explanation for these results could be that in cloning by somatic cell nuclear transfer the animals inherit maternal mitochondrial DNA and even though they have the same somatic DNA, the cloned pigs possess altering phenotypes due to the maternal mitochondrial DNA effect [9]. This raises the question of whether cloned animals are more suitable animal models than conventional non-cloned animals.

The heritable component of an individual and its effect on the microbial community have been investigated before in several human studies; in particular MZ twins have been investigated to minimize the genetic influence in order to get a better understanding of the role of obesity on gut microbiota [3]. When designing an experimental model for gut microbiota related studies, it is important to remove the large variability in the microbial community across individuals, making it necessary to use larger number of animals for valid statistical analysis and interpretation. Therefore, cloned animals could have the potential of becoming good models, by reducing the number of animals needed for an experimental study and providing a less variable population, however, more optimization is needed to improve the quality of the cloned animals.

In regard to obesity related gut microbiota, we did not observe any association between weight-gain and change in bacterial diversity, although there was more bacterial richness in obese pigs. Taken together; these results point to specific changes in the bacterial community over time in both the cloned and non-cloned control pigs.

To get a better profile of the gut microbial community in relation to obesity, we compared the relative abundance of the phyla *Bacteroidetes* and *Firmicutes* in the pigs from baseline and throughout the diet intervention period until endpoint. In the case of *Firmicutes,* we observed an increase in relative abundance of this phylum from baseline to endpoint, in both cloned and non-cloned pigs and found a positive correlation with *Firmicutes* and weight-gain. This increase in the abundance of the phylum *Firmicutes* with increase in weight is in agreement with observations made in other studies [15]. One study [29], point to a connection between alterations in energy intake and changes in gut microbiota such as increase in abundance of *Firmicutes*. Jumpertz and colleagues [21] found that a 20% increase in abundance of *Firmicutes* resulted in an increase in energy harvest corresponding to approximately 150 kilo calories. This suggests that the bloom in bacteria belonging to the phylum *Firmicutes* contributes to promotion of obesity and maintenance of the obese state.

The relative abundance of *Bacteroidetes* in the cloned pigs decreased continuously through the diet intervention period but then began steadily to increase until the animals were euthanized. The same was observed in the non-cloned control pig group and eventually the relative abundance of *Bacteroidetes* at endpoint was not different from baseline. This was unexpected, as previously it has been shown that obese subjects have less *Bacteroidetes* compared to their leaner counterparts [10,16,30]. Furthermore, one study on humans under a weight loss regiment showed [15] an increase in *Bacteroidetes*. One explanation to the observations made in our study could be that the bacteria belonging to phylum *Bacteroidetes* somehow adapt to the HF/high-caloric diet and their number at endpoint eventually reaches the values observed at baseline. Hildebrandt *et al.*[29] demonstrated a decrease in *Bacteroidetes* and an increase in *Firmicutes* in the gut microbiota of mice independent of obesity but in relation to HF diet in mice [29], while other studies point to the association of HF diet and the changes in abundance of *Firmicutes* in mice [4]. Together, these studies suggest that the changes in gut microbiota could be due to the HF/high caloric diet and not the state of obesity. Even though we found a positive relation between weight-gain and changes in the relative abundance of *Firmicutes,* we cannot exclude the possibility that the changes were also in relation to HF/high-caloric diet. Therefore, the gut microbiota could be a potential therapeutic target to fight obesity.

## Conclusion

Here we conclude that cloned pigs do not appear to have smaller inter-individual variation as compared to the sibling non-cloned pigs with regard to their gut microbiota, and because it is both time consuming and costly, they are not more suitable than conventional pigs for gut-microbiota-obesity related studies.

Our findings agree with the hypothesis that the diet-induced obesity is related to changes in the relative abundance of *Firmicutes* and *Bacteroidetes* and especially an increase in proportion of the bacteria belonging to the phyla *Firmicutes*. We also point to HF/high-caloric diet as a contributing factor that changes the gut microbial community. To our knowledge this is the first study that has investigated the effects of diet-induced obesity on gut-microbiota in cloned pigs. More investigation is needed to optimize the cloning of experimental animals which could eventually offer a more controlled experimental model.

## Competing interest

All authors declare no financial or any other competing interest.

## Authors’ contributions

MB, LM and RP designed the study experiments. RP carried out the experimental work, data and statistical analysis and wrote the manuscript. A.D.A performed the statistical analysis on T-RFLP Shannon-Weaver diversity and PCA and contributed to writing of the manuscript. JS designed and conducted the animal and the diet-intervention experiments. All authors read, corrected and approved the final manuscript.

## Supplementary Material

Additional file 1 **An overview of T-RFs (bp) in cloned and non-cloned pigs and possible bacterial taxonomy as estimated *in silico *through the MICA online database.** Click here for file

Additional file 2 **Correlation between weight gain and relative abundance of *Bacteroidetes * and *Firmicutes.*** Correlation between weight-gain and relative abundance of *Bacteroidetes* as calculated by Spearman correlation in cloned pigs (r= −0.33, *P*<0.04) and non-cloned control pigs and correlation between weight-gain and relative abundance of *Firmicutes* in cloned pigs (r= 0.37, *P*<0.02) and non-cloned control pigs (r=0.45, *P*<0.006). Each color represents a pig in that group i.e. pig 1 is indicated by a red dot and so on. Click here for file
