# Supplementary material for: Changes in the gut microbiota of cloned and non-cloned control pigs during development of obesity: gut microbiota during development of obesity in cloned pigs
Source: BMC Microbiol. 2013 Feb 7;13:30. doi: 10.1186/1471-2180-13-30 (PMC3610253; doi:10.1186/1471-2180-13-30)
Supplement: Additional file 1 — An overview of T-RFs (bp) in cloned and non-cloned pigs and possible bacterial taxonomy as estimated in silico through the MICA online database. [file 1471-2180-13-30-S1.docx]

**Additional file 1** An overview of T-RFs (bp) in cloned and non-cloned pigs and possible bacterial taxonomy as estimated *in silico* through the MICA online database.

| Controls | Clones | Possible T-RF *in silico* identification |
| --- | --- | --- |
| 62 | 62 | Actinobacteria |
|  | 91 | Bacteroidetes |
| 93 | 93 | Bacteroidetes |
| 95 | 95 | Unkown Bacterium |
|  | 96 | Not found in the database |
| 97 | 97 | Not found in the database |
| 98 | 98 | Not found in the database |
| 99 | 99 | Not found in the database |
|  | 100 | Unknown Bacterium |
| 101 | 101 | Unknown Bacterium |
| 102 | 102 | Bacteroidetes |
| 104 |  | Bacteroidetes |
|  | 170 | Unknown Bacterium |
|  | 188 | Unknown Bacterium |
|  | 189 | Firmicutes |
| 191 | 191 | Firmicutes |
| 192 | 192 | Firmicutes |
|  | 198 | Unknown bacteria |
| 208 |  | Proteobacteria |
| 218 |  | Unclassified bacterium |
| 228 |  | Firmicutes |
| 230 |  | Firmicutes |
| 231 | 231 | Firmicutes |
|  | 365 | Uncultured bacterium |
| 368 | 368 | Proteobacteria |
| 370 | 370 | Actinobacteria |
| 374 |  | Firmicutes |
| 379 |  | Uncultured bacterium |
| 386 | 386 | Uncultured bacterium |
|  | 389 | Firmicutes |
| 390 |  | Firmicutes |
|  | 405 | Firmicutes |
| 446 | 446 | Uncultured bacterium |
|  | 565 | Uncultured bacterium |
| 566 | 566 | Uncultured bacterium |
| 569 | 569 | Proteobacteria |
| 571 |  | Uncultured Proteobacteria |
| 574 | 574 | Uncultured bacterium |
| 585 | 585 | Firmicutes |
| 592 | 592 | Firmicutes |
| 594 | 594 | Firmicutes |
| 597 | 597 | Firmicutes |
|  | 632 | Unidentified bacteria |
|  | 635 | Not found in the database |
| 702 | 702 | Not found in the database |
|  | 736 | Not found in the database |
|  | 743 | Uncultured bacterium |
|  | 748 | Uncultured bacterium |
| 752 | 752 | Uncultured bacterium |
