# Supplementary figures and images for: Changes in the gut microbiota of cloned and non-cloned control pigs during development of obesity: gut microbiota during development of obesity in cloned pigs
Source: BMC Microbiol. 2013 Feb 7;13:30. doi: 10.1186/1471-2180-13-30 (PMC3610253; doi:10.1186/1471-2180-13-30)

Clones (*Bacteroidetes*)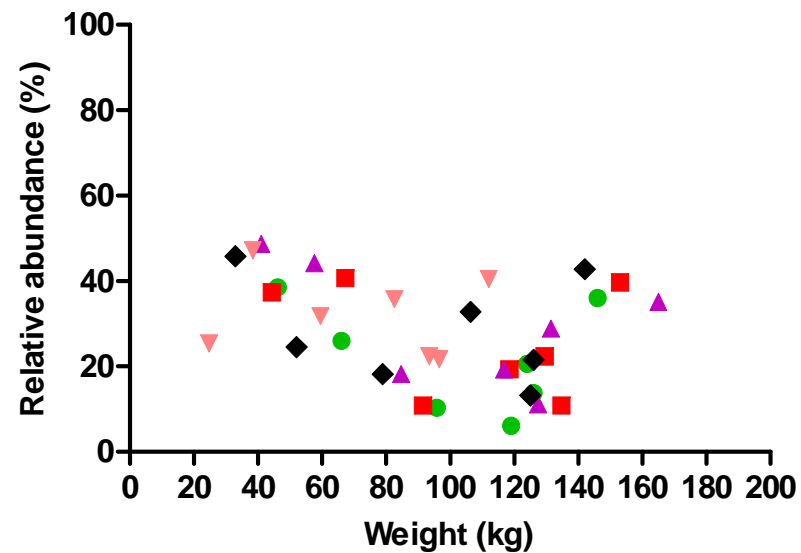Clones (*Firmicutes*)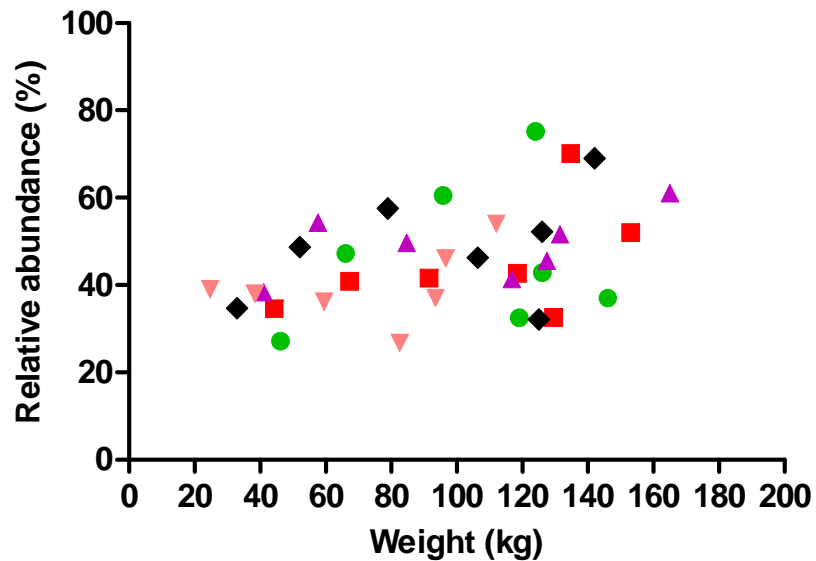Controls (*Bacteroidetes*)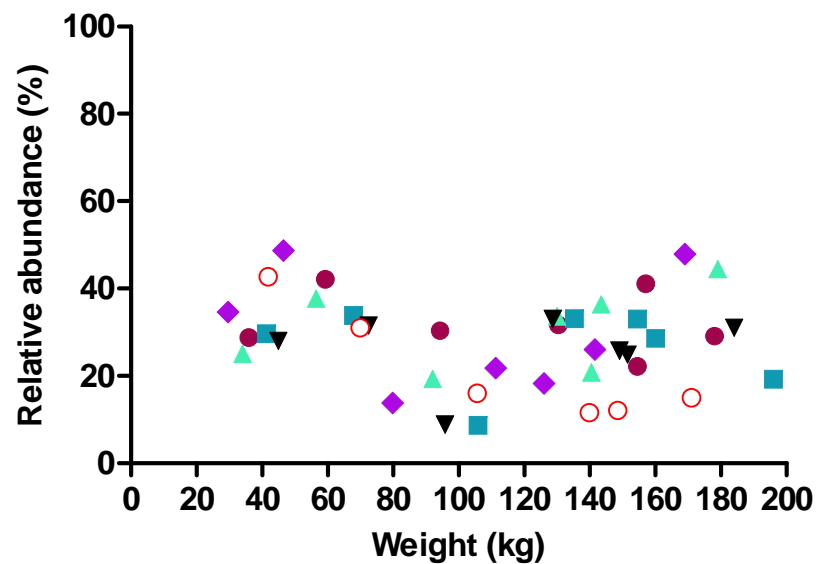Controls (*Firmicutes*)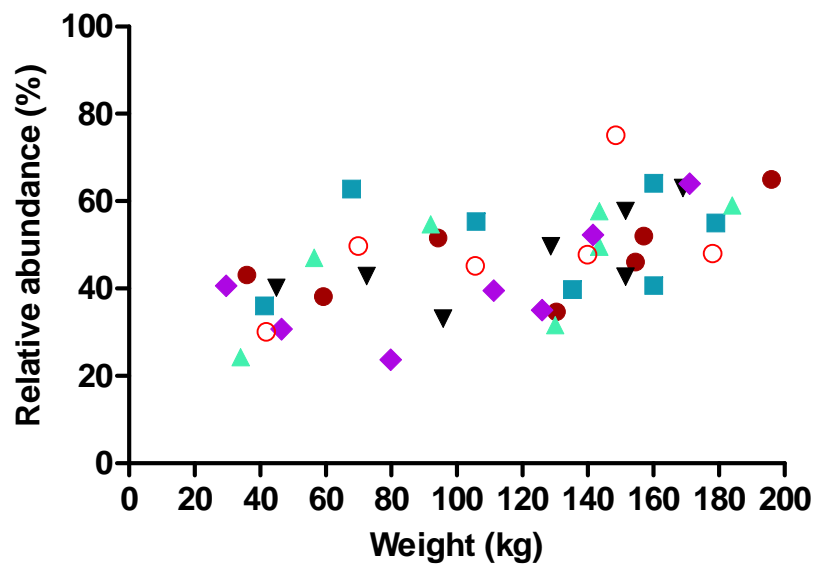

Supplement: Additional file 2 — Correlation between weight gain and relative abundance of Bacteroidetes and Firmicutes. Correlation between weight-gain and relative abundance of Bacteroidetes as calculated by Spearman correlation in cloned pigs (r= −0.33, P<0.04) and non-cloned control pigs and correlation between weight-gain and relative abundance of Firmicutes in cloned pigs (r= 0.37, P<0.02) and non-cloned control pigs (r=0.45, P<0.006). Each color represents a pig in that group i.e. pig 1 is indicated by a red dot and so on. [file 1471-2180-13-30-S2.pdf]
